# The prototypical UK blood donor, homophily and blood donation: Blood donors are like you, not me

**DOI:** 10.1111/vox.13731

**Published:** 2024-09-02

**Authors:** Eamonn Ferguson, Sarah Bowen, Richard Mills, Claire Reynolds, Katy Davison, Claire Lawrence, Roanna Maharaj, Chris Starmer, Abigail Barr, Tracy Williams, Mark Croucher, Susan R. Brailsford

**Affiliations:** ^1^ School of Psychology University of Nottingham; ^2^ National Institute for Health and Care Research Blood and Transplant Research Unit in Donor Health; ^3^ Behavioural Practice, Verian (formally Kantar Public); ^4^ NHS Blood and Transplant/UK Health Security Agency (UKHSA) Epidemiology Unit, NHSBT; ^5^ NHS Blood and Transplant/UK Health Security Agency Epidemiology Unit, UKHSA; ^6^ LawrencePsychAdvisory; ^7^ UK Thalassaemia Society; ^8^ School of Economics University of Nottingham; ^9^ Sickle Cell Society UK; ^10^ NHS Blood and Transplant, Donor Experience Services

**Keywords:** demography, equality, ethnicity, homophily, prototype, social class

## Abstract

**Background and Objectives:**

Homophily represents the extent to which people feel others are like them and encourages the uptake of activities they feel people like them do. Currently, there are no data on blood donor homophily with respect to (i) people's representation of the average prototypical UK blood donor and (ii) the degree of homophily with this prototype for current donors, non‐donors, groups blood services wish to encourage (ethnic minorities), those who are now eligible following policy changes (e.g., men‐who‐have‐sex‐with‐men: MSM) and recipients. We aim to fill these gaps in knowledge.

**Materials and Methods:**

We surveyed the UK general population MSM, long‐term blood recipients, current donors, non‐donors and ethnic minorities (*n* = 785) to assess perceptions of the prototypical donor in terms of ethnicity, age, gender, social class, educational level and political ideology. Homophily was indexed with respect to age, gender and ethnicity.

**Results:**

The prototypical UK blood donor is perceived as White, middle‐aged, middle‐class, college‐level educated and left‐wing. Current donors and MSM are more homophilous with this prototype, whereas recipients and ethnic minorities have the lowest homophily. Higher levels of homophily are associated with an increased likelihood of committing to donate.

**Conclusion:**

The prototype of the UK donor defined this as a White activity. This, in part, may explain why ethnic minorities are less likely to be donors. As well as traditional recruitment strategies, blood services need to consider broader structural changes such as the ethnic diversity of staff and co‐designing donor spaces with local communities.

**Highlights**

- The prototypical UK blood donor is White, middle‐aged, middle‐class, college‐level educated and left‐wing.
- Degree of homophily (the closeness of a person's perception of the prototypical UK blood donor to their own demography) predicts decisions to donate.
- Current donors and men‐who‐have‐sex‐with‐men are more homophilous with the blood donor prototype; ethnic minorities have the lowest homophily, with White people having the highest.

## INTRODUCTION

People are more likely to join groups, participate in sports, contribute to community initiatives/activities and seek healthcare if they feel that people who partake in those activities are similar to them [1, 2, 3]. This is called homophily [1, 3]. Homophily has important implications for donor services aiming to enhance diversity and equality in their donor panels by recruiting and retaining donors across more comprehensive ranges of ethnicity, sexuality and age [4, 5], Specifically, people who do not perceive themselves to be like—*homophilous* with—current donors are less likely to donate. This may, in part, explain why Black people and younger people are less represented in donor panels [4, 5]. Furthermore, following recent changes to United Kingdom (UK) donor policy men‐who‐have‐sex‐with‐men (MSM) are eligible to donate [6]. Thus, it is useful to know if MSM perceive themselves as homophilous to blood donors, as this is likely to encourage more MSM to donate. Therefore, knowledge of donor homophily for these groups is important for blood services to aid the development of inclusive strategies. Finally, the perspective of those with sickle cell and thalassaemia is critical. As long‐term recipients of blood treatment, efficacy is enhanced with well‐matched blood from ethnic minorities. Thus, these recipients may have concerns about their treatment if they do not see donors as homophilous for ethnicity.

Therefore, we explore how people in the UK define the prototypical blood donor across key demographic characteristics (e.g., age, sex, ethnicity), from the perspective of different stakeholders (blood donors, MSM, recipients of blood, people from ethnic minorities) and how donor homophily predicts active decisions to become a blood donor [3].

## BLOOD DONATION, HOMOPHILY, PROTOTYPE THEORY AND DONOR IDENTITY

Greater diversity of donors is beneficial both psychologically (e.g., increased well‐being) [7] and clinically (e.g., improved treatment of sickle cell disease: SCD) [4]. However, few new young people [5, 8] and members of ethnic minorities [4] donate blood. As an example, recruiting and retaining more Black donors will enhance the efficacy of treating Sickle Cell by better blood matching [4]. Furthermore, recent changes to UK donor selection policy, based on individualized sexual behaviour, mean that MSM can donate [6]. Again, MSM are more likely to decide to donate the greater their perceived homophily with current donors.

We argue that homophily is an additional structural barrier to donation. Indeed, many barriers to blood donation have been documented [9]; including psychological (e.g., fear of needles) and structural (e.g., convenience, location) factors that influence everyone [9], and some that are more likely to influence people from the Black community (e.g., distrust, fear of negative health effects, differential deferral) [10, 11, 12]. However, one major structural barrier, not previously explored with respect to sexuality, ethnicity and age, relates to how far potential donors perceive themselves as similar to the prototypical donor—*homophily* [1, 13]. Theoretical models are described below, highlighting why this is a potentially important driver/barrier to blood donor behaviour.

The prototype‐willingness model offers a dual‐process account of behaviour driven by a reactive emotional/heuristic and a planned decision‐making route [13]. The emotional/heuristic route encompasses the idea of behavioural prototypes: particular behaviours (e.g., blood donation) are associated with specific prototypes and the greater the degree of perceived similarity a person feels to the prototype, the more likely they are to perform that behaviour [13, 14].

Linked to prototypicality, is the concept of homophily. Homophily states that people are more likely to join groups/communities or prototypes to which they feel similar, in terms of both psychological *and* demographic characteristics [1, 3]. Conversely, people avoid behaviours/groups where homophily is low [2]. Arguably, if people perceive the typical donor as a member of a group with which they do not identify, they are less likely to donate blood.

Donor identity, which is a key driver of donor return behaviour [15, 16], arises not only as a function of donating per se [17] but also by identifying with similar other donors (prototype and homophily) [18]. This in‐group identity will reinforce the donor's self‐identity as a donor, encouraging return behaviour, which will ultimately perpetuate the current status quo and donor prototype [18]. Thus, there is a self‐reinforcing system whereby homophily enhances donor self‐identity, which in turn enhances return behaviour of homophilous people, which then further reinforces self‐identity.

Finally, there is a growing realization that ‘space’ is partly defined in terms of demography, including ethnicity, age, gender, social class and politics and that these characteristics influence who will be likely to enter these spaces [14, 19]. For example, if blood donors are perceived as being White, then ethnic minorities will be less willing to enter spaces where blood donation occurs.

## WHO ARE THE DONORS?

So, what are the current characteristics of voluntary blood donors? Regarding demography, in the UK, blood donors tend to be White in their late 30s to mid‐40s, with females slightly outweighing males [5]. Data from other countries indicate that blood donors are of higher socioeconomic and educational status and are educated to at least 18 years [20]. While there are no data on blood donors' political views, organ donors, who are also more likely to be blood donors, typically express a more politically left viewpoint [21]. If the prototype reflects these objective characteristics, people should view donors as equally likely to be male or female, of higher social status, educated, politically left, white and in their early 40s.

Therefore, in this article, we explore the perceived prototypical blood donor, calculate homophily scores for people from different cultural, social and health backgrounds to quantify their similarity to the prototypical donor and investigate whether those homophily scores predict decisions to donate.

## METHOD

### Sampling

Participants were recruited via (i) Prolific (https://www.prolific.com/about/) (18–23 November 2021), (ii) the UK Sickle Cell Society (23–29 November 2021) and (iii) UK Thalassaemia Society (22–29 November 2021). A two‐stage sampling process was adopted for the Prolific sample. An initial gender‐balanced UK adult sample was recruited, and the second was a UK adult sample of non‐heterosexual, non‐asexual identifying MSM. The samples were collected consecutively, and additional screening was performed to ensure no repeat recruitment. MSM were oversampled to explore awareness and beliefs about the (For the Assessment of Individualised Risk) FAIR project (not the focus of this paper). All respondents were paid £1.00 for participation, consistent with Prolific guidelines. The UK Sickle Cell Society sent the link to all their relevant social media channels and their registered members' email list and posted it on their dedicated blood donation awareness pages, ‘Give Blood, Spread Love, England.’ (https://www.instagram.com/givebloodspreadlove/). For the UK Thalassaemia Society, the link was distributed on all their relevant social media channels (Twitter, Facebook), their registered members' email list (there are 1600, including people with thalassemia, parents and doctors) and 4000 on their social media accounts. Responses were collected from 22 to 29 November 2021.

### The survey

The survey was programmed in Qualtrics (https://www.qualtrics.com/uk/). The key variables used in this paper are described below (see Supplementary File S1 for the full survey focus and sampling).

#### Demographics

Demographic information on age, gender, sexual orientation, ethnicity, religion and UK location was collected. Participants were coded as LGBTQ+ if they reported a sexual orientation other than heterosexual/straight *and/or* non‐binary gender identity. Participants were coded as MSM if they identified as bisexual, gay, queer, pansexual or bi‐curious and were male.

#### Donor history

All respondents were asked whether they had ever donated blood in the UK (Yes/No/ I'm not sure/ Prefer not to say) and were coded as *blood donors* if they responded ‘Yes’. Blood donors were subsequently asked (i) when they last donated blood (within the last 2 months/ 2 to 12 months ago/12 months to 2 years ago/Longer than 2 years/I cannot remember/Prefer not to say). *Non‐donors* are those who have never donated, *lapsed donors* have donated but not within the last 2 years, and *current donors* have donated in the last 2 years. This is a validated and reliable estimate of past donor behaviour [11, 22].

#### Prototypical donor

To assess what participants perceived a typical donor to be like in terms of demography, we asked, ‘In your mind, what does the ‘typical’ blood donor look like across the following demographic categories?’ They then selected one category for *age* (18–29, 30–44, 45+), using these categories because the proportion of donors aged 45 and over has increased in recent years (from 48.7% in 2018/19 to 51.1% in 2022/2023, NHSBT 2024). They also select one category for each of the following: *gender* (male, female); *ethnicity* (Asian, Black, Mixed, Other, White); *education level* (no‐qualification, General Certificate of Secondary Education (GCSE) or equivalent, A levels or equivalent, degree or equivalent); *social class* (working class, middle class, upper class) and *political affiliation* (left‐wing, right‐wing).

#### Homophily Index

To index homophily, we designate *π* = prototype demographic categorization and *σ* = person actual self‐ascribed demographic categorization. Then, in a specific dimension, if *π*–*σ* = 0, homophily, *η* = 1, else 0. Then, overall homophily, *Η* = ∑ (*η*). We calculated homophily scores using the demographic data available for both the respondents and their prototype judgements: age, gender and ethnicity. Thus, we have three dimension‐specific homophily scores each with a value 0 (=non‐similarity) or 1 (=the perceived donor categorization and participant categorization are the same). The total homophily scores, Η, range from 0 to 3, where 0 indicates that the respondent shares neither age group, gender, nor ethnicity with a prototype donor, and 3 indicates that the respondent shares all three. We applied unit weighting to each demographic characteristic when assessing the overall homophily score. While some demographic characteristics may have greater salience, there are no previous data in this domain to estimate or justify a specific weighting. Therefore, we chose unit weighting in this case.

#### Active commitment to donate blood

Evidence shows that an active commitment to donate is an extremely strong predictor of subsequent donations (Ferguson et al., 2023). As such, it is useful to identify predictors of making an active commitment to donate. To assess this, we stated:

In the UK, men can donate blood every 12 weeks, and women every 16 weeks. If you were to become a blood donor, would you expect to donate blood once or multiple times?

Participants then selected one of the following: Once, Multiple times, I'm not sure or prefer not to say. Selecting once or multiple times indicated an active decision to donate and selecting I'm not sure indicated hesitancy and indecision. This is a reliable index of future behaviour [23, 24].

#### Ethics

This survey study was approved by the School of Psychology, the University of Nottingham., Ethics Review Board (F1308) on the 15^th^ of November 2021.

#### Power estimates

A small effect size is observed for cognitive and emotional factors on emotions and donor behavioural propensity [23, 24, 25]. Thus, to achieve 0.80 power, with an *α* of 0.05, requires 332 participants.

## RESULTS

### Sample

In total, 804 participants were recruited; four respondents did not provide full informed consent, 11 dropped out after receiving the participant information sheet and 4 dropped out immediately after providing informed consent, giving a final sample of 785 observations. A Combined Patient Group (CPG) comprised participants who reported living with either thalassaemia or sickle cell.

Table 1 provides the sample characteristics (Supplementary File S2 and Table S1 provides a sample breakdown and representativeness analysis). Excluding the oversampling of MSM and the patient sample, the sample was younger (median 34) than the UK population in 2010 (median 40) and included more White people (89% vs 82%), but was broadly representative by location and gender. This pattern was the same for the full sample, except the oversampling of MSM increased the proportion of men in the sample.

**TABLE 1:** Summary descriptive statistics.

Demographics	Freq	Mean/%	SD	Min	Max	*n*
Age		35.77	12.77	18	81	779
Gender						
Male	518	66%		0	1	780
Female	251	32%		0	1	780
Non‐binary	11	1.5%		0	1	780
Prefer not to say	3	0.5%				
Sexual orientation						
LGBTQ+	328	42%		0	1	775
Straight	447	58%		0	1	775
MSM						
MSM	268	35%		0	1	767
Non‐MSM	499	65%		0	1	767
Ethnicity						
Asian	63	8%		0	1	772
Black	23	3%		0	1	772
Mixed	24	3%		0	1	772
Other	13	2%		0	1	772
White	649	84%		0	1	772
Location						
England	660	85%		0	1	781
Scotland	75	10%		0	1	781
Wales	32	4%		0	1	781
Northern Ireland	14	2%		0	1	781
Blood donation						
Non‐donors	546	70%		0	1	776
Lapsed donors	131	17%		0	1	776
Current donor	99	13%		0	1	776
Recipients of donated blood						
Recipient of donated blood/blood products	77	10%		0	1	768
Sickle cell	4	1%		0	1	785
Thalassaemia	36	5%		0	1	785
Ineligible to donate	138	18%		0	1	785
Friend/family member with sickle cell disease	27	4%		0	1	724
Friend/family member with thalassaemia	39	5%		0	1	710
Friend/family member who is a blood recipient	284	46%		0	1	616

Abbreviations: Freq, frequency; max, maximum; min, minimum; MSM, men‐who‐have‐sex‐with‐men.

### The Prototypical UK Blood Donor

Table 2 categorizes the prototypical donors as seen for the total sample, as well as by MSM, patients, current donors and ethnicity. Overall, the prototypical UK donor is perceived to be 30–44 years old, White, educated to A level (high school) or degree level, middle class and left‐wing. There is no clear perception that donors are more likely to be male or female.

**TABLE 2:** Prototypical donors as seen by sub‐groups.

	Responders sub‐groups									
	All	MSM	Patients	Current donors	White	Non‐White	Asian	Black	Mixed	Other
**Prototype categories**										
Sex										
Female	**404 (51.5)**	127 (47.4)	15 (37.5)	**55 (55.6)**	**343 (52.9)**	55 (45.2)	28 (48.3)	**14 (60.9)**	10 (41.7)	3 (23.1)
Male	381 (48.3)	**141 (52.6)**	**25 (62.5)**	44 (44.4)	306 (47.1)	**67 (54.8)**	**34 (51.7)**	9 (39.1)	**14 (58.3)**	**10 (76.9)**
*p* value	0.438	0.427	0.154	0.315	0.158	0.319	0.526	0.405	0.541	0.092
Age (years)										
18–29	235 (30.4)	86 (32.3)	8 (22.9)	29 (29.3)	177 (27.5)	**56 (47.5)**	**30 (50.8)**	**13 (56.5)**	9 (37.5)	4 (33.3)
30–44	**419 (54.3)**	**138 (51.9)**	**20 (57.1)**	**52 (52.5)**	**360 (56.0)**	51 (43.2)	25 (42.4)	8 (34.8)	**10 (41.7)**	**8 (66.6)**
45+	118 (15.3)	42 (15.8)	7 (20.0)	18 (18.2)	106 (16.5)	11 (9.2)	4 (6.8)	2 (8.7)	5 (20.8)	0 (0)
*p* value	**<0.001**	**<0.001**	**0.011**	**<0.001**	**<0.001**	**<0.001**	**<0.001**	**0.019**	0.417	0.248
Ethnicity										
Asian	12 (1.6)	0 (0)	1 (3.0)	1 (1.0)	0 (0.0)	12 (10.3)	12 (20.7)	0 (0.0)	0 (0.0)	0 (0.0)
Black	7 (0.9)	0 (0)	2 (6.1)	0 (0)	0 (0)	7 (6.0)	0 (0.0)	7 (31.8)	0 (0.0)	0 (0.0)
Mixed	49 (6.4)	16 (6.0)	5 (15.2)	3 (3.0)	32 (5.0)	14 (12.1)	5 (8.6)	1 (4.5)	8 (33.3)	0 (0.0)
Other	18 (2.3)	7 (2.6)	3 (9.1)	3 (3.0)	12 (1.9)	5 (4.3)	2 (3.4)	1 (4.5)	0 (0.0)	2 (16.7)
White	**681 (88.8)**	**243 (91.4)**	**22 (66.7)**	**92 (93)**	**596 (93.1)**	**78 (67.2)**	**39 (67.2)**	**13 (59.1)**	**16 (66.6)**	**10 (83.3)**
*p* value	**<0.001**	**<0.001**	**<0.001**	**<0.001**	**<0.001**	**<0.001**	**<0.001**	**<0.001**	0.102	**0.021**
Education										
No qualifications	11 (1.5)	3 (1.1)	2 (7.1)	1 (1.0)	10 (1.6)	1 (0.9)	0 (0.0)	1 (4.8)	0 (0.0)	0 (0.0)
GCSE or equivalent	120 (16.0)	45 (17.1)	2 (7.1)	17 (17.5)	108 (17.1)	11 (10.1)	5 (9.1)	2 (9.5)	3 (13.0)	1 (10.0)
A level or equivalent	**315 (42.1)**	**114 (43.3)**	8 (28.6)	**43 (44.3)**	**274 (43.8)**	38 (34.9)	22 (40.0)	6 (28.6)	6 (26.1)	4 (40.0)
Degree or equivalent	303 (40.5)	101 (38.4)	**16 (57.1)**	36 (37.1)	240 (38.0)	**59 (54.1)**	**28 (50.9)**	**12 (57.1)**	**14 (60.9)**	**5 (50.0)**
*p* value	**<0.001**	**<0.001**	**<0.001**	**<0.001**	**<0.001**	**<0.001**	**<0.001**	**0.003**	**0.015**	0.273
Social class										
Working class	214 (27.9)	66 (24.8)	6 (18.2)	23 (23.2)	177 (27.7)	35 (30.2)	15 (25.9)	7 (31.8)	11 (45.8)	2 (16.7)
Middle class	**541 (70.5)**	**198 (74.4)**	**27 (81.8)**	**73 (73.7)**	**465 (71.3)**	**76 (65.5)**	**40 (60.0)**	**14 (63.6)**	**12 (50.0)**	**10 (83.3)**
Upper class	12 (1.6)	2 (0.8)	0 (0.0)	3 (3.0)	7 (1.1)	5 (4.3)	3 (5.2)	1 (4.5)	1 (4.2)	0 (0.0)
*p* value	**<0.001**	**<0.001**	**<0.001**	**<0.001**	**<0.001**	**<0.001**	**<0.001**	**0.003**	**0.010**	**0.021**
Political ideology										
Left‐wing	**868 (84.7)**	**224 (85.2)**	**21 (72.4)**	**86 (88.7)**	**543 (85.6)**	**88 (80.0)**	**43 (76.8)**	**16 (76.2)**	**19 (82.6)**	**10 (100)**
Right‐wing	115 (15.3)	39 (14.8)	8 (27.6)	11 (11.3)	91 (14.4)	22 (20.0)	13 (23.2)	5 (23.8)	4 (17.4)	0 (0.0)
*p* value	**<0.001**	**<0.001**	**0.024**	**<0.001**	**<0.001**	**<0.001**	**<0.001**	**0.027**	**0.003**	**0.002**

*Note*: A binomial test was used for dichotomous variables and chi‐square for multi‐category variables within the demographic target category. The figures in blod indicate the largst number in that category.

Abbreviation: MSM, men‐who‐have‐sex‐with‐men. GCSE, General Certificate of Secondary Education

### Homophily

**FIGURE 1:**
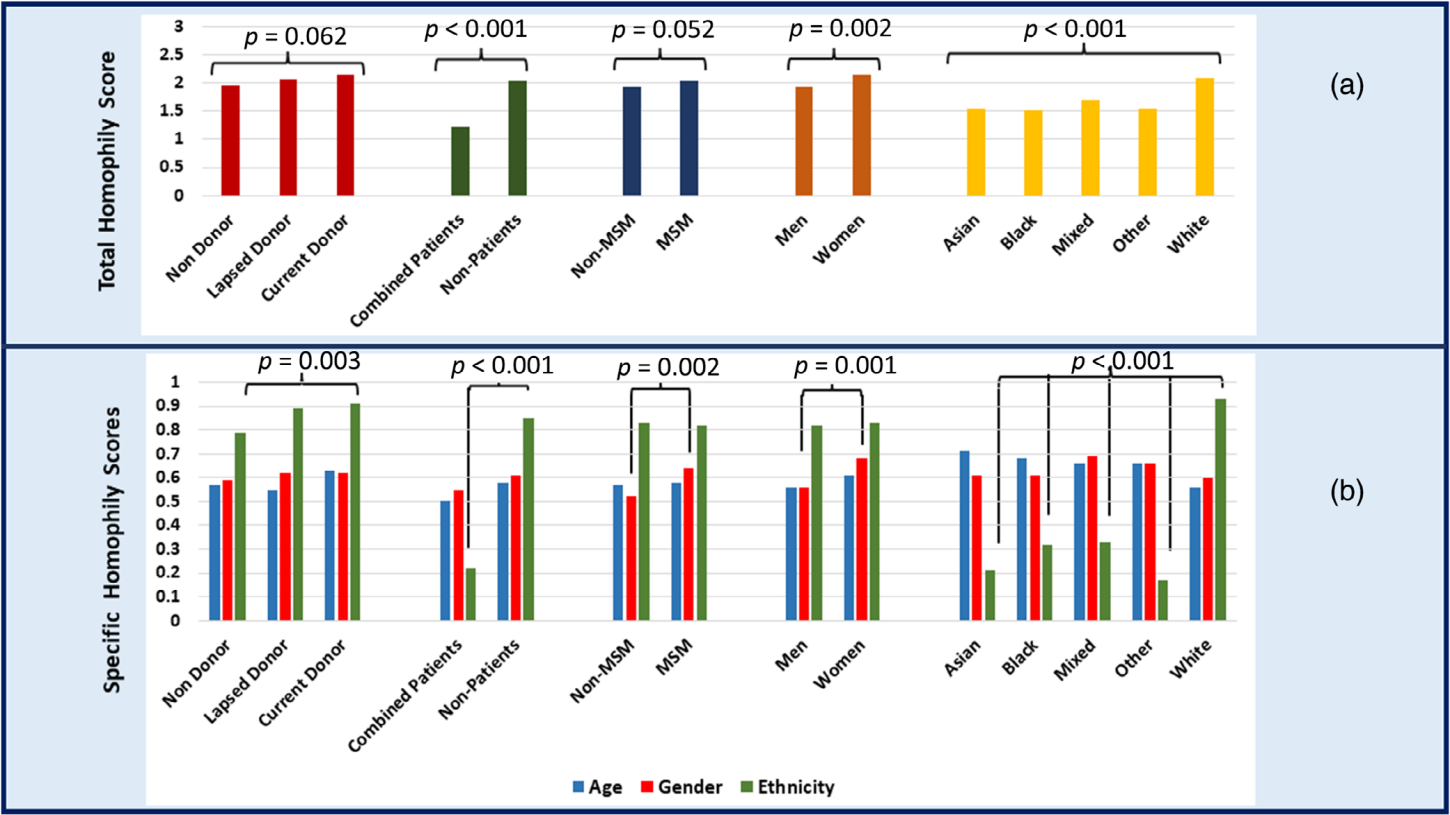
Homophily scores general (panel a) and specific (panel b) by sub‐groups. *p* = exact *p* value.

Figure 1 shows the homophily scores by sample characteristics (see Supplementary File S3 and Table S2 for exact figures for Figure 1). We see that this is 2 out of 3 for the overall sample. Current donors have the highest overall homophily score of 2.15 out of 3, significantly higher than non‐donors but similar to lapsed donors. This is driven by the ethnicity homophily score, in which current and lapsed donors have a higher average ethnicity homophily score and are thus more likely to perceive the prototypical donor's ethnicity as the same as their own ethnicity. Patients had the lowest homophily score of 1.22, which is significantly lower than non‐patients and, again, this is primarily related to ethnicity homophily. Patients view themselves as less similar in ethnicity to their perception of the prototypical donor. MSM had a higher homophily score (2.04) than non‐MSM, driven by the gender homophily. Thus, MSM see their gender (men) as similar to their perception of the gender of the prototypical donor. Women have a higher homophily score than men, which is also driven by the gender homophily scores, with women perceiving themselves as more similar to the prototypical donor in terms of gender. Homophily also varied by ethnicity, with Asian, Black, mixed and other ethnicities all having lower homophily scores than White participants.

### Predicting donation decisions

Seventy‐eight people said they would donate once, 293 many times, 72 were unsure and two preferred not to say. We explored, using a multi‐nominal regression model, the extent to which the overall homophily score predicts the active decision to make one or more donations compared to uncertainty about donating. The results show that a homophily score of two or three predicts an active decision to make more than one donation, compared with feeling uncertain about donating (Table 3: This effect is robust to the inclusion of demographic and prototype information as controls; see Supplementary File S4).

**TABLE 3:** Multinominal regression for active donation decisions on overall homophily scores in eligible non‐donors (*n* = 420).

	Coefficient (SE)	*z*	*p* value	95% CI	
				Lower	Upper
Uncertain					
Donate once					
Homophily score					
1	0.2336 (0.8316)	0.28	0.779	−1.3962	1.8634
2	0.4547 (0.8168)	0.56	0.578	−1.1462	2.0557
3	0.7646 (0.8206)	0.93	0.351	−0.8437	2.3729
Constant	−0.2877 (0.7638)	−0.38	0.706	−1.7846	1.2092
Donate many times					
Homophily score					
1	1.0371 (0.7194)	1.44	0.149	−2.4471	0.0373
2	1.4394 (0.7099)	2.03	0.043	0.0478	2.8301
3	1.4190 (0.7185)	1.97	0.048	0.0107	2.8274
Constant	0.2231 (0.6708)	0.33	0.739	−1.0916	1.5379

*Note*: Eligible non‐donors do not include recipients of blood. This is a multinomial regression model with ‘Uncertain about donation’ as the reference category and, within the homophily scores, zero is the reference category.

Abbreviations: CI, confidence interval.

## DISCUSSION

The prototypical UK blood donor is seen as 30–44 years old, White, educated to A level (high‐school) or degree level, middle class and politically left‐wing. We explored the degree of homophily with this prototype across a set of key stakeholders, including donors and non‐donors, to better understand the role of homophily with respect to donor retention (donors) and recruitment (non‐donors). Recruiting people from ethnic minorities is a major focus of many blood collection agencies; as such, we explored homophily from the perspective of a number of ethnic minorities (Asian, Black and Mixed). Recent policy changes in the UK (the FAIR project) and across the world with respect to individualized risk assessment of sexual behaviour mean that previous deferral policies for MSM no longer apply [6]. Therefore, we explored if MSM perceive the prototypical donor as like them. In general, greater homophily should be associated with a greater willingness to become or remain a donor. Finally, we explored the patient perspective from the vantage point of long‐term recipients of blood for those with sickle cell or thalassaemia. These recipients require multiple transfusions, and the efficacy of transfusions increases with well‐matched blood from ethnic minority donors. As sickle cell and thalassaemia are more prevalent in Black and Asian communities, lower homophily with the prototypical donor may lead to recipients' concerns about the efficacy of their current and future treatment.

Current donors perceive themselves as being most similar to the prototype donor, followed by MSM, with blood recipients being the least similar. People from ethnic minorities also have low homophily scores. As greater homophily increases the probability of making an active decision to be a repeat donor, the UK prototypical donor accurately reflects, and is likely driven by, the aggregate demographic profile of UK blood donors [26]. Perceptions of prototypical donors are associated with the decision to donate via the homophily score, with smaller perceived differences between a person's prototype and their own personal demography increasing their likelihood of donating.

While the perception that the prototypical UK blood donor is 30–44 years old, White, college‐educated, middle class and left‐wing reflects the demography of UK blood donors [26], this is not simply a reflection of the UK's wider demography, as there are demographic profiles for different philanthropic acts. For example, volunteers and those who donate money to charity tend to be older (65+ years), with an even distribution across ethnicity [27, 28, 29].

**FIGURE 2:**
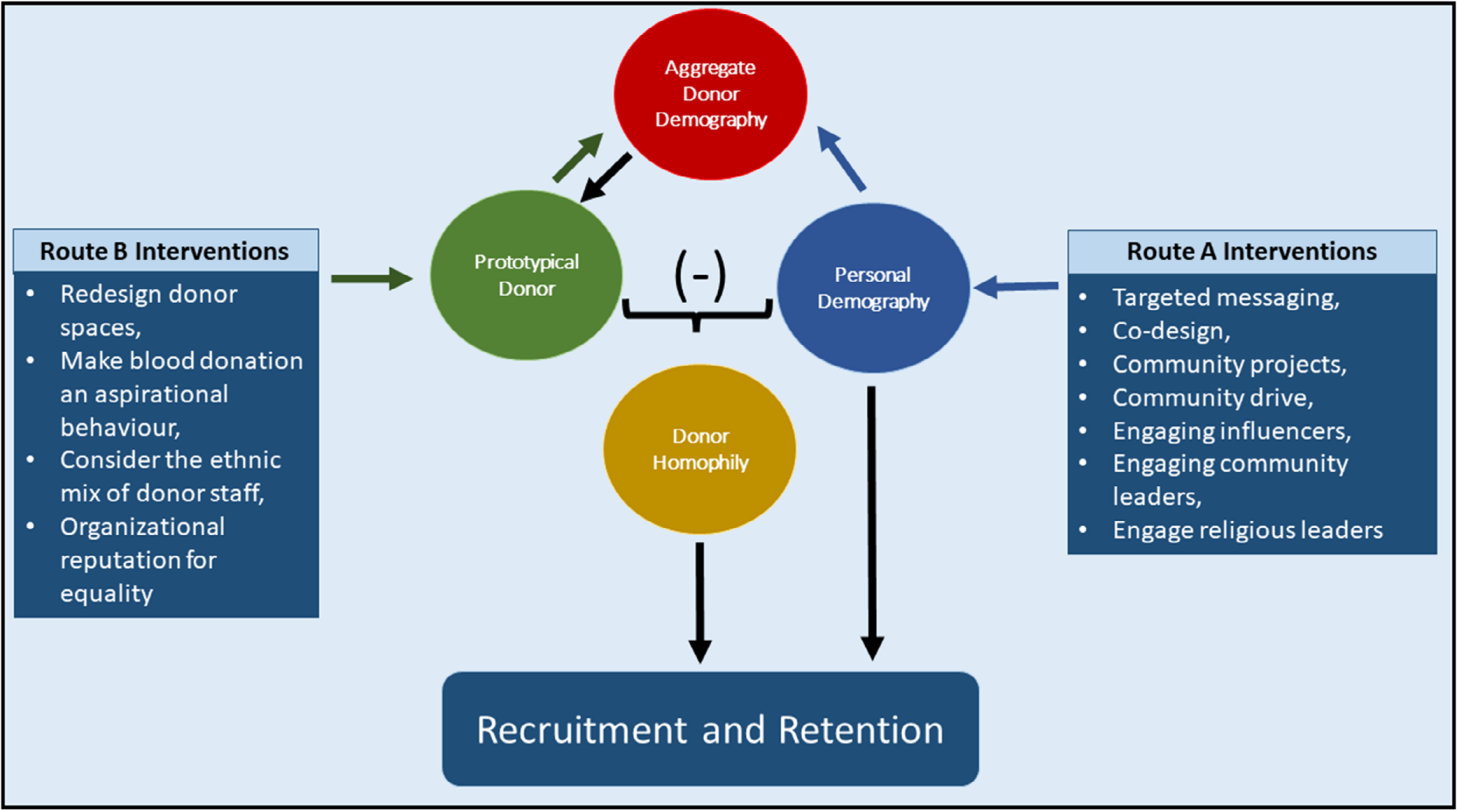
Theoretical and practical schema. The schema shows that homophily, defined as the difference between the perceived prototypical donor and the person's own demographic characteristics, drives donation decisions. This role for homophily highlights the dynamic relation between the prototypical donor and actual donor demography within a country. That is, actual demography predicts the prototype, but changing the perceived prototype (Route B) alters homophily and recruitment, altering the actual donor demography within a country and, thus, the prototype. Hence, there is a dynamic reinforcing link between the prototype and actual donor demography. This dynamic link can also be influenced by directly attracting a wider demography to donor panels (Route A).

Within the UK, White people constitute the largest ethnic group and, as such, many social, institutional and communal spaces become defined as White spaces. Nonetheless, there are spaces defined as Black and Asian, including clubs and cafes  [29, 30, 31, 32]. However, based on the prototypical donor, blood donation centres, like many UK institutions, are not. With that in mind, the perception of the prototypical donor may deter people from ethnic minorities and younger people. These are two groups blood services want to recruit [2, 5, 8]. One clear implication for blood services is that designing campaigns and strategies to change donor demography (Route A Interventions in Figure 2) [33] addresses only half the picture. Success with Route A interventions will, over time, change the aggregate donor demography and, ultimately, what the prototypical donor is perceived to be. However, interventions must also be considered to address how people perceive blood donation/donors (Route B Interventions in Figure 2). Fortunately, some evidence suggests prototypes can be malleable [34].

We initially consider what innovations are suggested by route A. Many campaigns and strategies have been implemented to recruit and retain more donors from ethnic minorities and younger age groups, and some have been successful [33]. As these have been reviewed and discussed at length, we focus on novel implications arising from knowing the UK prototypical donor.

Donors are seen as older, so blood donation is less likely to be perceived as relevant for young people [35], who are also less likely to have received a blood transfusion [36] or to know people who need a transfusion [37]. This implies the need to make the notion of blood donation salient for younger people. One way is to implement ‘cognitive time travel’ and have younger people consider their future selves and link to other future concerns important to younger generations, such as climate change [37].

The perception that the prototypical UK blood donor is middle class may be a previously unrecognized barrier to donors from working‐ and upper‐class people. Social class, especially within the UK, is a strong social force with respect to group formation, social identity and behaviour [38]. A drive for wider social class inclusion will likely impact greater ethnicity and educational inclusions, as these characteristics are geographically clustered and related [39]. Blood drives and campaigns generally focused across wider geographical and social areas may be worth considering.

A novel and interesting finding is the perception that the prototypical UK blood donor is left‐wing. Left‐wing ideology, compared with right‐wing ideology, is associated with increased compassion for others [40], which taps into wider associations of compassion, altruism and helping those in need [25]. Unfortunately, we do not know the current political ideology of UK blood donors. Without knowing this, it is difficult to propose effective strategies. However, having politicians from all ideologies jointly endorse blood donation as a compassionate act may encourage wider diversity of donors.

Prior research has identified a wide set of barriers to blood donation including psychological concerns (anxiety, phobia of needles and blood), structural issues (inconvenience, location and time), as well as issues specific to minorities, such as prejudice and differential deferral [9, 10, 11, 12]. We show that homophily should be added as a structural and specific barrier.

Below, we explore how this barrier may be addressed, focusing on the types of intervention suggested by route B. As blood donors are both perceived as White and the majority *are* White, the perception of blood donation as a White activity in a White space will act as a barrier to ethnic minorities becoming blood donors [1].

Potential solutions could involve locating blood centres in geographical areas where the density of ethnic minorities is high. This could be enhanced further by increasing the diversity of donor centre staff. Ideally, blood donor centres should be co‐designed with members of the local ethnic minority communities to make these spaces more culturally relevant, welcoming and familiar. NHSBT's work with the new co‐designed Brixton Blood Centre in London is an excellent example.

The donor centre location is also important in terms of how political ideology influences blood donor behaviour. What is important concerning political ideology and blood donor behaviour is not the *absolute* ideology (left‐wing, right‐wing) but rather partisanship, with individuals less likely to donate blood when their political ideology is very different from the representative political ideology of their area [41]. Specifically, those who perceive themselves as political outliers are less likely to donate blood. Therefore, political ideology is an important consideration for blood services. Again, this is another reason for blood donation centres to consider *where* their donor centres are placed and the importance of co‐designing with the local demography and developing community‐based partnerships and funding schemes.

Donor services need to change the perception of blood donation as an exclusively middle‐aged activity, especially if they wish to recruit younger donors [42]. One possible strategy is to normalize and represent blood donation as a positive, socially normative activity through social media (e.g., Instagram, TikTok, BeReal or Snapchat posts). Blood donation could be presented as an aspirational and community‐building activity for young people and made relevant to them.

This is a primarily descriptive study, and we make no claims of causality. We look at the prototype as an antecedent to recruitment but acknowledge that there are many complexities to donor recruitment. However, the implications of these results underscore the importance of the blood donor prototype and homophily, which should now be considered in future work.

The study has some limitations. The sample was not representative by ethnicity and age; however, the consistency of the findings by age, gender and ethnicity supports the contention that this did not affect the results. We also acknowledge that the age categories were not uniform, which may have contributed to the prototypical age effect being middle age; future research would benefit from incorporating a more comprehensive range of evenly distributed age bands. We assessed *active decisions to commit to donate blood* as this is a key predictor of actual donation [24]. As such, we did not assess directly if people were completely *un*willing to donate, and this should be explored in future studies. Finally, causality needs to be explored and the use of instrumental variable models, propensity score matching and Directed Acyclic Graphs (DAG)s can all be considered [43, 44].

## CONFLICT OF INTEREST STATEMENT

The authors declare no conflicts of interest.

## Supporting information


**Data S1.** Supporting Information.

## Data Availability

The data that support the findings of this study are available from the corresponding author upon reasonable request.
