# Supplementary material for: The prototypical UK blood donor, homophily and blood donation: Blood donors are like you, not me
Source: Vox Sang. 2024 Sep 2;119(12):1223–33. doi: 10.1111/vox.13731 (PMC11634441; doi:10.1111/vox.13731)
Supplement: Supplementary file 1 — Data S1. Supporting Information. [file VOX-119-1223-s001.docx]

**Supplementary Files for The Prototypical UK Blood Donor, Homophily, and Blood Donation: Blood Donors Are Like You, Not Me.**

Table of Contents

[Supplementary Files S1: Survey Design and Sampling 2](#_Toc172720436)

[***Figure S1***. *Study DesiDesign Flow and Sampling* 2](#_Toc172720437)

[Supplementary Files S2: Sub-Sample Details 3](#_Toc172720438)

[***Table S1***. *Sub-Samples* 3](#_Toc172720439)

[***Representativeness*** 3](#_Toc172720440)

[Supplementary File S3: Homophily Scores by Sample Sub-Groups 4](#_Toc172720441)

[***Table S2***. *Homophily Scores by Dimension Across Sub-Groups* 4](#_Toc172720442)

[Supplementary Files S4: Multinominal Regression with Controls 5](#_Toc172720443)

[***Table S3***. *Multinominal Regression Predicting Donation Decisions by Homophily Controlling for Sample Demography (age, gender, ethnicity and MSM) and Protoitypcal Donor Perceptions (Social Class, Educational Level and Political Ideology)* 5](#_Toc172720444)

# Supplementary Files S1: Survey Design and Sampling

Figure S1 below details the study sampling flow, the focus of the questions and the sampling procedures.

## ***Figure S1***. *Study DesiDesign Flow and Sampling*


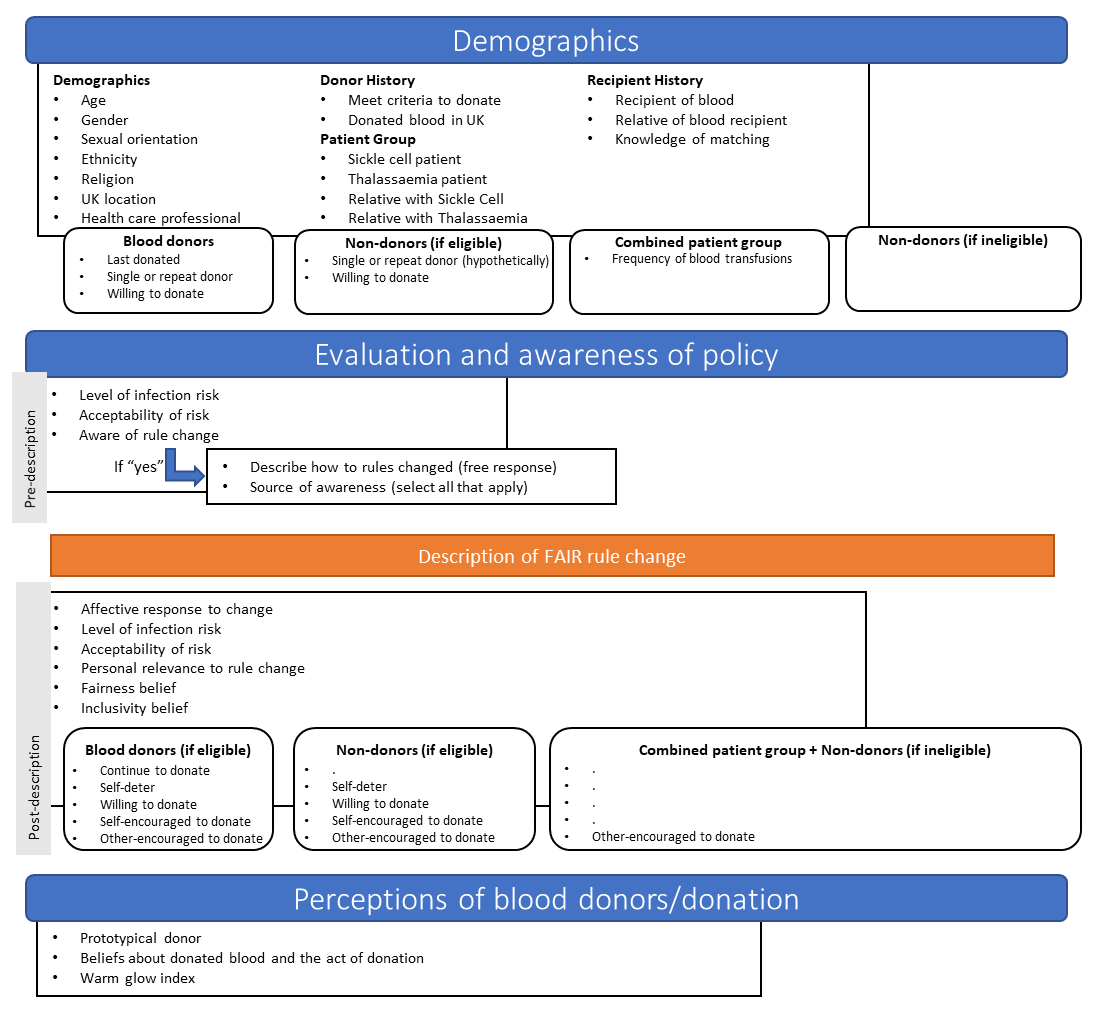


# Supplementary Files S2: Sub-Sample Details

Tabke S1 below details the study sub-samples.

## ***Table S1***. *Sub-Samples*

|  | Prolific UK  gender-balanced | Prolific UK  MSM | Sickle cell Society | Thalassaemia UK | Total |
| --- | --- | --- | --- | --- | --- |
| **Ineligible to donate** | 45 | 48 | 6 | 39 | 138 |
| Sickle cell | 0 | 0 | 4 | 0 | 4 |
| Thalassaemia | 1 | 0 | 0 | 35 | 36 |
| **Combined patient group** | 1 | 0 | 4 | 35 | 40 |
|  |  |  |  |  |  |
| **Non-donors** | 272 | 238 | 5 | 37 | 552 |
| **Blood donors** | 129 | 87 | 5 | 12 | 233 |
| Regular donors (< 2 yrs) | 54 | 38 | 5 | 2 | 99 |
| Lapsed donors (2+ yrs) | 74 | 48 | 9 | 0 | 131 |
|  |  |  |  |  |  |
| Heterosexual/straight | 340 | 57 | 9 | 41 | 447 |
| LGBTQ+ | 56 | 265 | 1 | 6 | 328 |
| MSM | 18 | 248 | 0 | 2 | 268 |
|  |  |  |  |  |  |

## ***Representativeness***

The study was conducted in 2021. At that time, the UK population had a median age of 40 years; 51% were female (49% male), 84% resided in England, 8% in Scotland, 5% in Wales and 3% in Northern Ireland. 9.3% were Asian, 4% were Black, 2.9% were Mixed Ethnicity, 2.1% were Other, and 82% were White. Let's examine our sample excluding the oversampling of MSM and the samples from UK Thalassaemia and UK Sickle Cell Society. That sample has a median age of 34 (so is slightly younger), 49% are female (51% male), so broadly representative, 82% resided in England, 10% in Scotland, 5% in Wales and 2.5% in Northern Ireland, so again broadly representative. In this sample, 5.2 % were Asian, 3% were Black, 1.8% were Mixed Ethnicity, 1.0% were Other, and 89% were White, so slightly over sampling White people. Let's now examine the full sample of 785 people (including MSM and the patient samples). We have a median age of 33, so again lower than the UK population, 67% are now male, but this reflects the over-sampling of MSM: 84% resided in England, 10% in Scotland, 4% in Wales and 2% in Northern Ireland, so again broadly representative. In this sample, 8.0 % were Asian, 3% were Black, 3.1% were Mixed Ethnicity, 1.7% were Other, and 84% were White, so slightly oversampling White people.

# Supplementary File S3: Homophily Scores by Sample Sub-Groups

Table S2 details the homophily scores, standard deviation and sample size by study sub-groups

## ***Table S2***. *Homophily Scores by Dimension Across Sub-Groups*

|  | **Total Homophily score** | | **Age homophily** | | **Gender homophily** | | **Ethnicity homophily** | |
| --- | --- | --- | --- | --- | --- | --- | --- | --- |
|  | Mean (sd) n | Test and p-value | Mean (sd) n | Test and p-value | Mean (sd) n | Test and p-value | Mean (sd) n | Test and p-value |
| All | 2.00 (0.86) N = 740 |  | 0.58 (0.49) N = 767 |  | 0.60 (0.49) N = 769 |  | 0.82 (0.38) N = 757 |  |
| Non-Donor  vs Lapsed Donor.  vs Current Donor | 1.96 (0.85) n = 514  2.07 (0.85) n = 121  2.15 (0.89) n = 98 | **Z = 5.56 p =.062** | 0.57(0.49) n = 533  0.55 (0.50) n = 127  0.63 (0.48) n = 98 | Χ^2^= 1.61 p = .448 | 0.59(0.49) n = 534  0.62 (0.49) n = 129  0.62 (0.49) n = 99 | Χ^2^= 0.41, p = .813 | 0.79(0.40) n = 526  0.89 (0.32) n = 124  0.91 (0299) n = 99 | **Χ^2^= 11.52 p = .003** |
| Combined patient group vs non-patients | 1.22 (0.99) n = 31  2.04 (0.84) n = 709 | **Z = 4.50 p <.001** | 0.50 (0.51) n = 32  0.58 (0.49) n = 735 | Χ^2^= 0.82, p = .364 | 0.55 (0.50) n = 40  0.61 (0.49) n = 729 | Χ^2^= 0.50, p = .478 | 0.22 (0.42) n = 32  0.85 (0.35) n = 725 | **Χ^2^= 85.47 p <.001** |
| Non-MSN vs  MSM | 1.92 (0.85) n = 264  2.04 (0.87) n = 474 | **Z = 1.94 p = .052** | 0.57 (0.49) n = 266  0.58 (0.49) n = 485 | Χ^2^= 0.02, p = .879 | 0.52 (0.50) n =268  0.64 (0.48) n = 497 | **Χ^2^= 9.73 p = .002** | 0.83 (0.37) n = 264  0.82 (0.38) n = 479 | Χ^2^= 0.19, p = .661 |
| Men vs  Women | 1.94 (0.86) n = 501  2.15 (0.84) n = 239 | **Z = 3.10 p = .002** | 0.56 (0.50) n = 509  0.61 (0.49) n = 242 | Χ^2^= 1.93, p = .165 | 0.56 (0.50) n = 518  0.68 (0.46) n = 251 | **Χ^2^= 10.44 p = .001** | 0.82 (0.38) n = 502  0.83 (0.38) n = 241 | Χ^2^= 0.58, p = .810 |
| *Ethnicity group* |  | **H = 31.32 p <.001** |  | Χ^2^= 7.08, p = .132 |  | Χ^2^= 1.12, p = .891 |  | **Χ^2^= 321.35, p <.001** |
| Asian | 1.53 (0.88) 58 |  | 0.71 (0. 46) 58 |  | 0.61 (0.49) 62 |  | 0.21 (0.41) 58 |  |
| Black | 1.52(1.03) 21 |  | 0.68 (0.47) 22 |  | 0.61 (0.50) 23 |  | 0.32 (0.48) 22 |  |
| Mixed | 1.69 (1.06) 23 |  | 0.66 (0.48) 24 |  | 0.69 (0.47) 23 |  | 0.33 (0.48) 24 |  |
| Other | 1.54 (1.04) 11 |  | 0.66 (0.49) 12 |  | 0.66 (0.49) 12 |  | 0.17 (0.39) 12 |  |
| White | 2.09 (0.21) 626 |  | 0.56 (0.50) 641 |  | 0.60 (0.49) 637 |  | 0.93 (0.25) 640 |  |

# Supplementary Files S4: Multinominal Regression with Controls

## ***Table S3***. *Multinominal Regression Predicting Donation Decisions by Homophily Controlling for Sample Demography (age, gender, ethnicity and MSM) and Protoitypcal Donor Perceptions (Social Class, Educational Level and Political Ideology)*

| Active Decision To Donate in the Future | Coefficient (Std. err.) | P = | [95% conf. | interval] |
| --- | --- | --- | --- | --- |
| Uncertain About Donating | (base outcome) |  |  |  |
| **Would Donate Once** |  |  |  |  |
| Homophily |  |  |  |  |
| 1 | 0.5040801 (0.9002688) | .576 | -1.260414 | 2.268574 |
| 2 | 0.8957522 (0.9106909) | .325 | -0.889169 | 2.680674 |
| 3 | 1.11758 (0.9342874) | .232 | -.0713589 | 2.94875 |
| Sample Gender (female) | 0.3645357 (0.4869985) | .454 | -0.589964 | 1.319035 |
| Sample Ethnicity (Non-White) | 1.001817 (0.5227793) | .055 | -0.022811 | 2.026446 |
| Sample Age Categories |  |  |  |  |
| 30 -44 | 0.1121064 (0.4013202) | .780 | -.6744667 | 0.8986795 |
| 45+ | -0.3326602 (0.5413246) | .539 | -1.393637 | 0.7283166 |
| Sample MSM | 0.2625171 (0.4072557) | .519 | -0.535689 | 1.060724 |
| Donor Prototype Social Class |  |  |  |  |
| Middle class | .1345372 (0.432454) | .756 | -0.713057 | 0.9821315 |
| Upper class | -.0226877 1.354322 | .987 | -2.67711 | 2.631734 |
| Donor Prototype Political Ideology |  |  |  |  |
| Right-wing | 0.5084342 (0.5068328) | .316 | -.4849398 | 1.501808 |
| Donor Prototype Educational Level |  |  |  |  |
| GCSEs or equivalent | -1.304435 (1.263804) | .302 | -3.781445 | 1.172575 |
| A-levels or equivalent | -.09553108 (1.233498) | .439 | -3.372923 | 1.462301 |
| Degree or equivalent | -1.093076 (1.25129) | .382 | -3.54556 | 1.359407 |
| Constant | -0.0767805 (1.501773) | .959 | -3.020201 | 2.86664 |
|  |  |  |  |  |
| Would Diomate Many Times |  |  |  |  |
| Homophily |  |  |  |  |
| 1 | 0.9994883 (0.7570852) | .187 | -0.484371 | 2.483348 |
| **2** | **1.536183 (0.7676505)** | **.045** | **.031616** | **3.040751** |
| 3 | 1.390759 (0.7893656) | .078 | -.1563697 | 2.937887 |
| Sample Gender (female) | 0.6444279 (0.3992523) | .107 | -.1380922 | 1.426948 |
| Sample Ethnicity (Non-White) | 0.3806913 (0.4679279) | .416 | -.5364305 | 1.297813 |
| Sample Age Categories |  |  |  |  |
| 30 -44 | -0.0473019 (0.3366405) | .888 | -0.707105 | 0.6125012 |
| 45+ | -0.0252978 (0.4194464) | .952 | -0.847398 | 0.796802 |
| Sample MSM | .00018085 (0.3326927) | .996 | -.6502572 | .6538741 |
| Donor Prototype Social Class |  |  |  |  |
| Middle class | 0.2502421 (0.3600531) | .487 | -0.455449 | 0.9559332 |
| Upper class | 0.0817681 (1.175119) | .945 | -2.221423 | 2.384959 |
| Donor Prototype Political Ideology |  |  |  |  |
| Right-wing | 0.0135263 (0.4425407) | .976 | -0.853837 | 0.8808901 |
| Donor Prototype Educational Level |  |  |  |  |
| GCSEs or equivalent | 0.351306 (1.299874) | .787 | -2.1964 | 2.899012 |
| A-levels or equivalent | 0.8777551 (1.284762) | .494 | -1.640332 | 3.395842 |
| Degree or equivalent | 0.7778693 (1.29644) | .549 | -1.763106 | 3.318845 |
| Constant | -0.8787214 (1.472636) | .551 | -3.765035 | 2.007592 |
